# Profiling *FLT3* Mutations in Mexican Acute Myeloid Leukemia Pediatric Patients: Impact on Overall Survival

**DOI:** 10.3389/fped.2020.00586

**Published:** 2020-09-16

**Authors:** Carolina Molina Garay, Karol Carrillo Sánchez, Luis Leonardo Flores Lagunes, Marco Jiménez Olivares, Anallely Muñoz Rivas, Beatríz Eugenia Villegas Torres, Hilario Flores Aguilar, Juan Carlos Núñez Enríquez, Elva Jiménez Hernández, Vilma Carolina Bekker Méndez, José Refugio Torres Nava, Janet Flores Lujano, Jorge Alfonso Martín Trejo, Minerva Mata Rocha, Aurora Medina Sansón, Laura Eugenia Espinoza Hernández, José Gabriel Peñaloza Gonzalez, Rosa Martha Espinosa Elizondo, Luz Victoria Flores Villegas, Raquel Amador Sanchez, Maria Luisa Pérez Saldívar, Omar Alejandro Sepúlveda Robles, Haydeé Rosas Vargas, Angélica Rangel López, María Lilia Domínguez López, Ethel Awilda García Latorre, Elba Reyes Maldonado, Patricia Galindo Delgado, Juan Manuel Mejía Aranguré, Carmen Alaez Verson

**Affiliations:** ^1^Laboratorio de Diagnóstico Genómico, Instituto Nacional de Medicina Genómica (Inmegen), Mexico City, Mexico; ^2^Instituto de Diagnóstico y Referencia Epidemiológicos (InDRE), Mexico City, Mexico; ^3^Unidad de Investigación Médica en Epidemiología Clínica, UMAE Hospital de Pediatría, Centro Médico Nacional “Siglo XXI”, Instituto Mexicano del Seguro Social (IMSS), Mexico City, Mexico; ^4^Servicio de Hematología Pediátrica, Hospital General “Gaudencio González Garza”, Centro Médico Nacional (CMN) “La Raza”, Instituto Mexicano del Seguro Social (IMSS), Mexico City, Mexico; ^5^Unidad de Investigación Médica en Inmunología e Infectología, Hospital de Infectología “Dr. Daniel Méndez Hernández”, “La Raza”, Instituto Mexicano del Seguro Social (IMSS), Mexico City, Mexico; ^6^Servicio de Oncología, Hospital Pediátrico de Moctezuma, Secretaria de Salud del D.F., Mexico City, Mexico; ^7^Servicio de Hematología Pediátrica, UMAE Hospital de Pediatría, Centro Médico Nacional (CMN) “Siglo XXI”, Instituto Mexicano del Seguro Social (IMSS), Mexico City, Mexico; ^8^Servicio de Hemato-Oncología, Hospital Infantil de México Federico Gómez, Secretaria de Salud (SSa), Mexico City, Mexico; ^9^Servicio de Onco-Pediatría, Hospital Juárez de México, Secretaria de Salud (SSa), Mexico City, Mexico; ^10^Servicio de Hematología Pediátrica, Hospital General de México, Secretaria de Salud (SSa), Mexico City, Mexico; ^11^Servicio de Hematología Pediátrica, Centro Médico Nacional (CMN) “20 de Noviembre”, Instituto de Seguridad Social al Servicio de los Trabajadores del Estado (ISSSTE), Mexico City, Mexico; ^12^Hospital General Regional No. 1 “Carlos McGregor Sánchez Navarro”, Instituto Mexicano del Seguro Social (IMSS), Mexico City, Mexico; ^13^Unidad de Investigación Médica en Genética Humana, Unidad Médica de Alta Especialidad (UMAE) Hospital de Pediatría, Centro Médico Nacional (CMN) “Siglo XXI”, Instituto Mexicano del Seguro Social (IMSS), Mexico City, Mexico; ^14^Coordinación de Investigación en Salud, Instituto Mexicano del Seguro Social (IMSS), Mexico City, Mexico; ^15^Escuela Nacional de Ciencias Biológicas (ENCB), Instituto Politécnico Nacional (IPN), Mexico City, Mexico; ^16^Hospital Central Sur de Alta Especialidad de Pemex, Mexico City, Mexico

**Keywords:** *FLT3*, pediatric, Mexican, AML, survival, risk-stratification

## Abstract

**Background:** Acute myeloid leukemia (AML) is the second most frequent leukemia in childhood. The *FLT3* gene participates in hematopoietic stem cell proliferation. *FLT3* mutations are recurrent in AML and influence prognosis. In Mexican pediatric AML patients, *FLT3* mutational profile, and their clinical impact have not been evaluated.

**Aim of the study:** This study aimed to identify the profile of *FLT3* mutations in pediatric patients with *de novo* AML and to assess their possible influence on overall survival (OS) and other clinical features.

**Methods:** Massive parallel target sequencing of *FLT3* was performed in 80 patients.

**Results:** *FLT3* mutations [internal tandem duplication (ITD) or tyrosine kinase domain (TKD)] were identified in 24% of them. OS was significantly lower in *FLT3*^POS^ cases than in *FLT3*^NEG^ (*p* = 0.03). The average OS for *FLT3*^POS^ was 1.2 vs. 2.2 years in *FLT3*^NEG^. There were no significant differences in the children's sex, age, percentage of blasts in bone marrow aspirate, or white blood cell count in peripheral blood at diagnosis between both groups. No differences were identified stratifying by the mutational load (high > 0.4) or type of mutation. The negative effect of *FLT3* mutations was also observed in patients with acute promyelocytic leukemia (APL).

**Conclusions:** *FLT3* mutational profile is described in Mexican pediatric AML patients for the first time. Mutated *FLT3* negatively impacts the outcome of AML patients, even considering the APL group. The clinical benefit from treatment with tyrosine kinase inhibitors in the *FLT3*^POS^ pediatric patients needs to be assessed in clinical trials. *FLT3* testing may contribute to better risk stratification in our pediatric AML patients.

## Introduction

Genomic investigations of acute myeloid leukemia (AML) have demonstrated that several genes are recurrently mutated, leading to the identification of new biomarkers. Genetic screening of molecular abnormalities plays a significant role in prognostic categorization and definition of treatment strategies in AML (1, 2). *FLT3* is a class III family receptor tyrosine kinase expressed in hematopoietic stem cells and has essential roles in cell survival and proliferation. *FLT3* gene mutations are among the most frequently observed in AML, which occurs in approximately one-third of patients with *de novo* AML. They have been associated with the clinical prognosis, treatment response, and survival of patients (3).

Two types of *FLT3* activating mutations have been identified in AML. Internal tandem duplications (ITDs) affect the juxtamembrane (JM) domain or tyrosine kinase domain 1 (TKD1) of the *FLT3* receptor and point mutation preset in the TKD2 (4). Both types of mutations promote ligand-independent auto-phosphorylation and constitutive activation of the receptor. This unregulated activation impairs normal hematopoiesis and contributes to leukemogenesis (5).

ITD mutations are the most frequent and are considered drivers in AML. At diagnosis, the *FLT3*–ITD clone may possess a growth advantage. This clone becomes dominant at relapse through a clonal expansion process (6, 7). Higher relapse rates and more reduced survival have been associated with *FLT3*–ITD in previous studies, largely depending on the allelic ratio (AR) of the mutations and the presence of mutations in other genes (8). The clinical impact of *FLT3*–TKD mutations is less clear (9).

*FLT3* genetic testing, detecting both ITD and TKD mutations, is recommended by the National Comprehensive Cancer Network (10) and European Leukemia Net (3) for diagnostic workup of patients with AML, to identify those who may benefit from different targeted treatment options. *FLT3* screening is not routinely performed in Mexico. Epidemiological data about the *FLT3* mutational profile and its clinical impact in Mexican AML patients are minimal. Studies including a small number of *de novo* and secondary AML patients (children and adults) have reported a prevalence between 13 and 20% of *FLT3* mutation using polymerase chain reaction (PCR)-based technologies (11).

The landscape of somatic mutations in pediatric AML was shown to be markedly different from adults, suggesting different biology and etiology for adult and childhood AML (12). These findings stress the importance of analyzing the mutational profile and evaluate the clinical impact of genetic alteration separately in both groups of patients. The purpose of this study was to assess the prevalence and the influence of *FLT3* mutations on the outcome of pediatric AML *de novo* patients. AML is one of the main types of cancer in Mexican children; its incidence has a trend toward an increase in the last year, and mortality is higher than in developed countries despite that the same chemotherapy protocols are used (13).

## Materials and Methods

### Population

Eighty patients with *de novo* AML, coming from eight different public hospitals, were included. Patients were diagnosed between March 2010 and March 2018. AML diagnosis was performed at each institution using bone marrow aspirate and immunophenotype. The bone marrow samples analyzed in the present study were obtained at the time of the diagnosis, before treatment initiation, and submitted to the Mexican Inter-Institutional Group for Identifying Childhood Leukemia Causes in Mexico City.

A predefined set of data was collected from medical charts, including the child's sex, age, white blood cell (WBC) count in peripheral blood, and percentage of blasts in the bone marrow at diagnosis, French-American-British (FAB) classification, and treatment protocol.

The ethics and scientific review boards of the National Institute of Genomic Medicine, Mexico City, Mexico, approved this study (document number 28-2015-1). All human samples and clinical information were approved for its use in the present study. The child's parents signed the informed consent obtained in accordance with the Declaration of Helsinki.

### DNA Extraction

DNA was extracted using Maxwell® 16 Blood DNA Purification Kit (Promega, Madison, WI, USA) according to the manufacturer's recommendations. The purity and concentration of the DNA samples were measured using NanoDrop 1000 spectrophotometer (Thermo Fisher Scientific, Waltham, MA, USA) and Qubit fluorometer (Thermo Fisher Scientific, Waltham, MA, USA).

### Next-Generation Sequencing

Sequencing of the exons 13–15 and 20 of the *FLT3* gene was performed using the multigene panel “Myeloid solution” by SOPHiA Genetics (Sophia Genetics SA, Saint Sulpice, Switzerland). Library preparation and sequencing were performed according to the manufacturer's protocol. Capture-base enriched libraries were pooled (12 indexed libraries per pool) and sequenced on a MiSeq system using version 3 chemistry (Illumina, San Diego CA, USA). Sequencing data were analyzed with the Sophia DDM® software version 5.2.7.1 (Sophia Genetics SA, Saint Sulpice, Switzerland). Sequencing deep was higher than 1,000 × for all the target regions.

Variant fraction (VF) was computed by dividing the number of mutation-supporting reads by the total number of reads at the mutation site. AR was calculated by dividing the number of reads corresponding to the mutation by the number of reads according to the wild-type allele in the mutation site.

### Statistical Analysis

The comparison of proportions between different groups was made using chi^2^ or two-sided Fisher's exact test for dichotomic variables, when appropriate. A non-parametric Mann–Whitney *U-*test was used for continuous variables, *p* < 0.05 was considered as statistically significant. All calculations were performed using the SPSS software package, SPSS Version 21 (Chicago, IL, USA).

The Kaplan–Meier method (14) was used for assessing overall survival (OS). The log-rank test was used to evaluate differences between survival distributions with a 95% confidence interval (CI). The OS time was calculated from the day of diagnosis confirmation until either the last follow-up or patient's death from any cause. Patients who did not experience an event were censored at the time of the last follow-up. Those who did not attend a follow-up were censored at their date of the last known contact.

## Results

### Demographic and Biological Characteristics of the Patients

The demographic, clinical, and main biological features of the patients are shown in Table 1. Fifty-five percent of the cases were male. The median age at diagnosis was 9.3 (range 0.4–17.5) years; the age at diagnosis was very similar in both genders. Acute promyelocytic leukemia (APL), M3, was the most prevalent subtype (36.3%), followed by the M2 (33.8%). M0, M5, and M6 were the less common subtypes. APL seems to occur more frequently in Hispanic populations than in other ethnic groups (15).

**Table 1:** Demographic and biological characteristics of the patients.

**Gender**		***N =* 80**	**%**
	Male	44	55
	Female	36	45
**Median age at Dx**		**Years**	**Range**
	Male	9.3	(1.2–16.6)
	Female	9.25	(0.4–17.5)
	Total	9.3	(0.4–17.5)
**Median BM blast at Dx**		**%**	**Range**
		80	(23–100)
**WBC at Dx/mm**^**3**^		***N=*** **80**	**%**
	≤ 11,000	31	38.8
	>11,000–100,000	35	43.8
	>100,000	14	17.5
**Median of overall survival (years)**		**N (%)**	**Mean**
	Overall survival	79 (100%)	1.95
	Survival ≤ 1 year	30 (37.9%)	0.35
	Survival > 1 year	49 (62.1%)	2.93
**FAB subtypes**		***N=*** **80**	**%**
	M0	1	1.3
	M1	9	11.3
	M2	27	33.8
	M3	29	36.3
	M4	12	15.0
	M5	1	1.3
	M6	1	1.3

*Dx, diagnosis; BM, bone marrow; WBC, white blood cells; FAB, French-American-British*.

Almost 38% of the patients do not survive more than 1 year after diagnosis. The median OS in the study population was 1.95 years.

Patients were treated according to one of the following four protocols: BFM-1998, BFM-2001 (Berlin-Frankfurt-Münster 1998 and 2001), NOPHO-AML93 (Nordic Society of Pediatric Hematology and Oncology), or PETHEMA-APL-05 (Spanish Program of Treatments in Hematology). Treatment information is summarized in Supplementary Table 1. Noteworthy, all APL patients were treated according to the PETHEMA-APL-05 protocol. For the M2 group, BFM-1998, NOPHO-AML93, and BFM-2001 were used, while for M4 and M1, only BFM-1998, and NOPHO-AML-93 were applied. None of the patients received allogeneic or autologous bone marrow transplant.

Since chemotherapeutic drugs affect outcomes, we assess the impact of the treatment protocol in the OS (see Supplementary Figures 1A,B). No significant differences were found among patients treated with NOPHO-AML93, BFM-2001, or BFM-1998, although those patients receiving NOPHO-AML93 seem to have the best long-term outcome while BFM1998 the worst. Patients treated with these protocols were considered as a single group for comparison with the PETHEMA-APL-05 treated group. OS was significantly higher in APL patients in contrast to those children from other FAB subtypes and treated with any different therapeutic combination (Supplementary Figure 1B).

### *FLT3* Somatic Mutation Profile in Mexican Pediatric Acute Myeloid Leukemia Patients

Mutations in *FLT3* were identified in 23.7% (19/80) of the patients, being more frequent in females (52.6%). The mutational profile is shown in Table 2. ITD mutations were present in 52.6% (10/19) of the *FLT3*^POS^ cases, with VFs ranging from 2.4 to 64.9%. The range of affected amino acids expanded from position 584 to 613. In one patient, in-frame deletion of amino acid 600 in the JM domain was observed, without any insertions or duplications of nucleotides. This patient was included in the ITD group since the mutation may affect the functioning of the JM domain.

**Table 2:** Mutational profile of *FLT3* in pediatric AML patients.

**Patient ID**	**BM Blast %**	**Protein domain**	**NM_ cDNA**	**Protein consequence**	**VF%**	**AR**	**Coding consequence**	**COSMIC ID**
M158	72	JM	c.1751_1795dup	p.Ser584_Glu598dup	10.1	0.11	Inframe_45	
M149	68	JM	c.1758_1787dup	p.Asp586_Arg595dup	36.3	0.57	Inframe_30	COSM19948
M136	77	JM	c.1780_1800dup	p.Phe594_Asp600dup	40.2	0.67	Inframe_21	COSM19953
M186	98	JM	c.1782_1829dup	p.Phe594_Asn609dup	23.8	0.31	Inframe_48	
M180	92	JM	c.1784_1804dup	p.Arg595_Leu601dup	34.1	0.52	Inframe_21	COSM19883
M163^#^	70	JM	c.1798_1800delGAT	p.Asp600del	3.5	0.04	Inframe_3	
M183^&^	98	JM	c.1816_1817ins21	p.Phe605_Pro606ins7	30.2	0.43	Inframe_21	
M191	ND	JM-TK	c.1835_1836ins33	p.Leu601_Glu611dup	2.3	0.02	Inframe_33	
M126	65	TK	c.1831_1832ins60	p.Leu610_Glu611ins20	37.8	0.61	Inframe_60	
M169	96	TK	c.1836_1837ins111	p.Phe612_Gly613ins37	64.9	1.85	Inframe_111	
M172	90	TK	c.2508_2510delCAT	p.Ile836del	36.3	0.57	Inframe_3	COSM797
M124	71	TK	c.2510_2512delTGA	p.Met837del	1.7	0.02	Inframe_3	
M104	60	TK	c.2503G > T	p.Asp835Tyr	42.8	0.75	Missense	COSM783
M144	87	TK	c.2503G > T	p.Asp835Tyr	3.7	0.04	Missense	COSM783
M148	94	TK	c.2503G > T	p.Asp835Tyr	38.4	0.62	Missense	COSM783
M117	100	TK	c.2503G > C	p.Asp835His	25.2	0.34	Missense	COSM785
M188	ND	TK	c.2503G > C	p.Asp835His	1.6	0.02	Missense	COSM785
M134	45	TK	c.2516A > G	p.Asp839Gly	23.4	0.31	Missense	COSM1166729
M113	70	TK	c.2525A > C	p.Tyr842Ser	26.1	0.35	Missense	

*JM, juxtamembrane domain (amino acids 564–609); TK, tyrosine kinase domain (amino acid 610–943), according to (PF07714); VF, variant fraction; AR, allelic ratio*.

# *M163 a second mutation in TKD was identified (c.2503G > T, p.Asp835Tyr, VF = 1.4%)*.

& *M183 a second ITD mutation was identified (c.1759_1815dup, p.Asn587_Phe605dup, VF = 1.9%)*.

**Figure 1:**
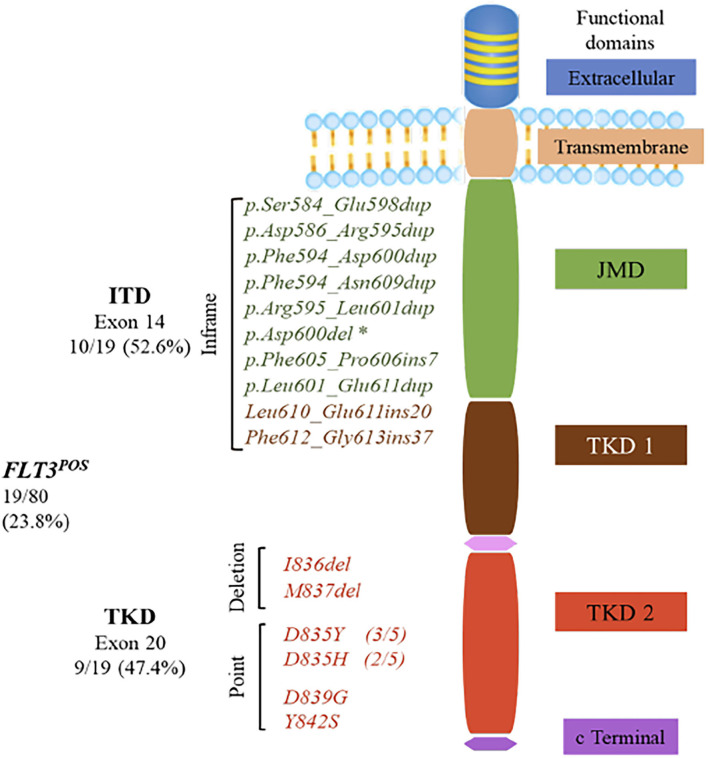
FMS-like tyrosine kinase 3 (*FLT3*) gene encodes a 993-amino-acid protein, consisting of five functional domains: an immunoglobulin-like extracellular domain, a transmembrane domain, a juxtamembrane domain (JMD), one interrupted tyrosine kinase domain (TKD), and a small C-terminal domain. Internal tandem duplications (ITDs) were the most common mutations in the *FLT3*^POS^ patients with insertions from 21 to 111 bp that mainly occurred in JMD, although two ITDs were found in the β1 sheet of TKD1. *Asp600del was positioned in exon 14 of the JMD, but it is not considered as an ITD, because no nucleotide duplication occurred. TKD activating mutations were located within the activation loop of the TKD2. Most of them were single-nucleotide variations resulting in amino acid changes at positions 835, 839, and 842. Two activating mutations caused by deletions were also identified in the TKD2.

Missense or small deletion mutations in the TKD region were present in 47.4% (9/19) of the *FLT3*^POS^ patients with VFs ranging from 1.6 to 42.8%. Changes in position 835 of the TKD domain were the most frequent. Two different amino acid changes were observed: p.Asp835Tyr in three cases and p.Asp835His in two. Additionally, single nucleotide variations affecting positions 839 and 842 of TKD were observed in one patient each. Two in-frame deletions affecting amino acid 836 and 837 were also found. A second *FLT3* mutation, at a lower VF, was identified in two patients (M183 and M163) (Table 2, Figure 1).

### Distribution of Demographic and Biological Features Between *FLT3* Positive and Negative Patients

Age at diagnosis, sex, percentage of blasts in bone marrow, and FAB subtype distribution was compared between *FLT3*^POS^ and negative patients. The results are displayed in Table 3.

**Table 3:** Distribution of demographic and biological features between *FLT3*^POS^ and negative patients.

		**FLT3**^****POS****^		**FLT3**^****NEG****^	
**Gender**		***N***	**%**	***N***	**%**
	Male	9	47.4	35	57.4
	Female	10	52.6	26	42.6
**Median age at Dx**		**Year**	**Range**	**Year**	**Range**
	Male	8.3	(1.2–15.8)	9.58	(1.7–16.6)
	Female	7.7	(0.5–16.7)	9.83	(0.4–17.5)
	Total	8.0	(0.5–16.7)	9.69	(0.4–17.5)
**Median BM blast at Dx**		**%**	**Range**	**%**	**Range**
		77	(45–100)	80	(14–100)
**WBC at Dx/mm**^**3**^		***N***	**%**	***N***	**%**
	≤ 11,000	8	42.1	23	37.7
	>11,000–100,000	6	31.6	29	47.5
	>100,000	5	26.3	9	14.8
**Median of overall Survival**		***N***	**Year**	***N***	**Year**
	Overall survival	19	1.17	60	2.2
	Survival ≤ 1 year	10	0.20	20	0.44
	Survival > 1 year	9	2.24	40	3.09
**FAB subtypes**		***N***	**%**	***N***	**%**
	M0	0	0.0	1	1.6
	M1	1	5.3	8	13.1
	M2	6	31.6	21	34.4
	M3	9	47.4	20	32.8
	M4	2	10.5	10	16.4
	M5	0	0.0	1	1.6
	M6	1	5.3	0	0.0

*Dx, diagnosis; BM, bone marrow; WBC, white blood cells; FAB, French-American-British*.

No significant differences were found. However, the median age at diagnosis was lower in the *FLT3*^POS^ group. When it was stratified by gender, *FLT3*^POS^ female patients were 2.1 years younger at diagnosis than *FLT3*^NEG^ females. In male *FLT3*^POS^ patients, the disease was diagnosed 0.4 years earlier than in the *FLT3*^NEG^ negative. None of these differences reached statistical significance.

### Analysis of the Association Between *FLT3* Mutation and Overall Survival

**Figure 2:**
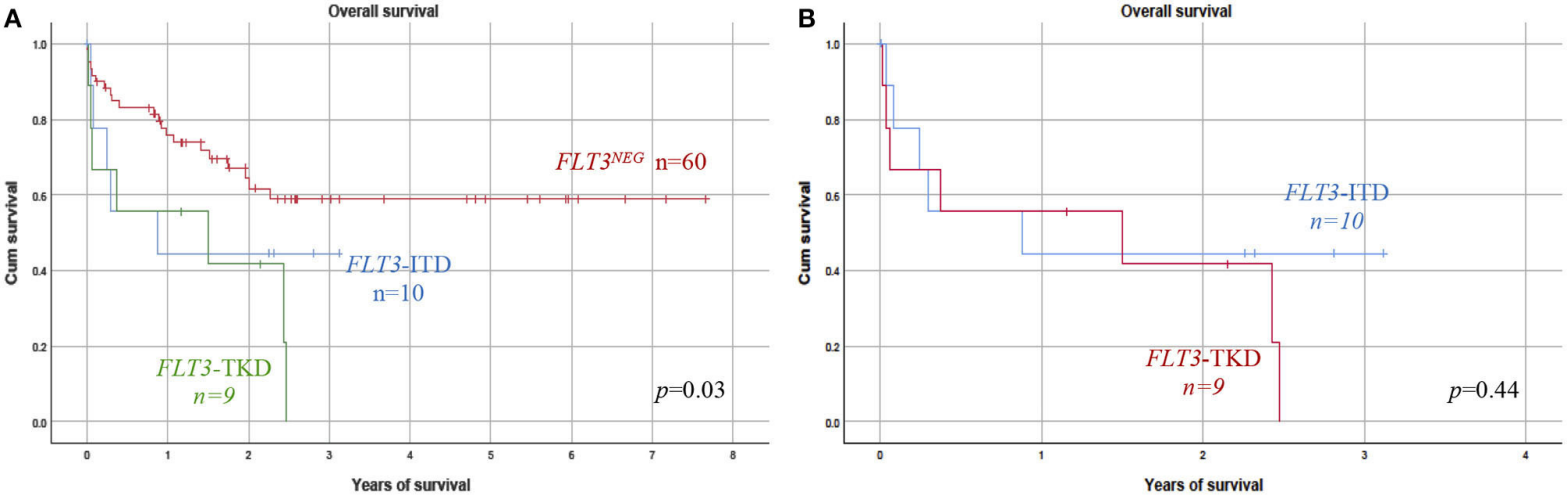
Prognosis impact of *FLT3* mutation. **(A)** Overall survival (OS) was analyzed considering *FLT3*–ITD or *FLT3*–TKD patients vs. the *FLT3*^NEG^ negative group. *FLT3*^POS^ patients (TKD or ITD) showed a significantly reduced OS as compared with those without *FLT3* mutation. **(B)** No significant difference in OS was found between *FLT3*–ITD and TKD groups of patients. ITD, internal tandem duplication; TKD, tyrosine kinase domain.

OS results, according to the *FLT3* status, are shown in Figures 2A,B. The OS was significantly decreased among patients with *FLT3* mutation (Figure 2A). The median of OS time was 1.2 (0.05–3.1) years in the *FLT3*^POS^ vs. 2.2 (0.05–7.6) years, in negative children (Table 3). When ITD or TKD mutations were considered separated (Figure 2B), no differences were observed between survival curves from ITD or TKD positive patients vs. *FLT3*^NEG^ patients. The OS analysis was calculated with 79 patients due to the date for the last contact or death was not available in one patient.

Since the allelic burden of the *FLT3* mutation has been shown to influence the outcome in adults, we stratified both *FLT3*–ITD and *FLT3*^POS^ (the type of mutation) patients according to their AR, using 0.4 as the cutoff value (16). The mutational burden did not influence OS, considering the *FLT3*–ITD group or the whole *FLT3*^POS^ group.

The impact of *FLT3* mutations was evaluated in the APL(M3) group of patients. The results are shown in the Supplementary Figure 2. APL-*FLT3*^POS^ patients had a significantly lower global survival than the APL-*FLT3*^NEG^ group. The OS of APL-*FLT3*^POS^ was similar to the OS observed for other FAB subtypes grouped (*p* = 0.014).

## Discussion

Mutational profiling of patients with AML is part of routine diagnostic workups for both *de novo* and relapsed AML patients in centers from developed countries. In Mexico, the *FLT3* mutational profile is not routinely performed in most public institutions; therefore, little epidemiological data exist about prevalence and heterogeneity of *FLT3* mutations and their influence on clinical evolution in both AML adult and pediatric patients.

This analysis of *de novo* AML cases represents the most extensive cohort of Mexican children and adolescents analyzed for *FLT3* mutational status. Eighty pediatric *de novo* AML patients, diagnosed between March 2010 and March 2018, were examined. The use of next-generation sequencing technology provides additional information about the length, position, and sequence of the identified mutations, revealing details about the structural variability of ITD and TKD mutations and the presence of additional clones.

*FLT3*–ITD or TKD mutations combined were present in 24% of the studied samples. To the best of our knowledge, there is no other study analyzing only Mexican AML children. Data about *FLT3* mutation are scarce for Latin-American AML pediatric patients. The reported prevalence in Costa Rica and Argentina is lower than in our patients (14.3% in 14 patients and 15.2% in 92 patients, respectively) (17). The results from Brazil are very similar to those from Mexico, with 23.3% reported in an extensive collaborative analysis, including a large number of pediatric patients (18).

More extensive analysis has been conducted in pediatric AML patients from the USA. In 2006, 19% of *FLT3*^POS^ cases (*FLT3*–ITD with 12% and *FLT3*-TKD with 6.7%) were detected in a group of 630 *de novo* AML pediatric patients, belonging to the Children's Cancer Group. The authors suggested that, in general, the frequency of mutated *FLT3* is 15% lower in the pediatric population than in adults (19). However, in 2015, Tarlock et al. (20) reported a prevalence of 28% of *FLT3*^POS^ cases in a group of 799 pediatric AML patients (16.02% with ITDs, 4.6% with TKDs, and 7.6% with novel mutation).

Differences in sensitivity between the *FLT3* detection methods used could be contributing to the lower prevalence of mutation observed in some of the previous studies compared with ours. Molecular methods based on PCR were employed in all of them. These methods can provide the length, but not the exact position or the sequence of the insertion, and have lower sensitivity than massive parallel sequencing technology. Mutations with meager allelic fraction could be missed by PCR-based methodologies. Interestingly, in this study, five patients had a VF lower than 4%. If these patients are removed from the prevalence calculation, the frequency of *FLT3* mutation turns to be only 17.5%.

*FLT3* mutations have been reported with a higher prevalence in adults than in children, although children have a mutational profile not observed in adults (12, 20). The prevalence in adults with AML ranges from ~20% (21) to 30% (22, 23). Reports from Europe, Japan, and China have shown a prevalence of 27.7% (24), 24.9% (23), and 21.23% (21), respectively.

The prevalence of *FLT3* mutation seems to be lower in adult AML Hispanic patients than in other groups, although technical limitations may have contributed to the observed differences (22). Ruiz Arguelles et al. reported 13% in 31 patients, while Arana Trejo et al. found 15% of *FLT3* mutated in 21 patients; both studies were done in Mexican patients (11). A collaborative study, including patients from referral centers in Mexico and Colombia, reported only 20% of *FLT3*^POS^ patients. They analyzed 138 patients with *de novo* or secondary AML (11). This study also included the patients previously reported by Arana Trejo and Ruiz Arguelles (11). In AML adults from Argentina, Sánchez et al. (25) reported 20.6%, while in Brazil, 26.3% was found (26).

Although the frequency of *FLT3* mutation was higher in the female group, no statistically significant difference in gender distribution was observed, similar to what has been found in most series (18). No association was seen between age at diagnosis, WBC, or blast percentage at bone marrow and the presence of *FLT3* mutations.

ITD mutation was present in 52.6% (10/19) of the patients. The length of the alteration varies from 21 to 111 bp, being 21 bp, the only size observed in more than one patient. The size of the duplicated region ranges from 15 to 174 bp (19), but up to 400 bp has been previously reported (3, 27).

In all cases, *FLT3*–ITD occurred in multiples of three nucleotides, preserving the reading frame of the transcript, as has been observed in other studies (3). Evaluation of the role of ITD size in clinical prognosis has opposite results. Some authors showed that increasing ITD size was associated with an adverse outcome (28), while others found no association (29). The number of patients in our study was too small to perform this analysis.

Furthermore, it has been suggested that ITD mutations always affect at least one amino acid residue from codon 591–597, with codon 597 being the most duplicated (30, 31). We do not confirm this observation. In our series, ITD mutations expand from codon 584–613. ITDs involving codons 591–597 were present only in 50% of the patients with this type of mutation. Only four of them had the amino acid 597 included in the duplication. For the other 50% of the ITD patients, the mutation affected codons 600–613.

Most of the ITD mutations were located only at the JM domain (6/9 patients). However, in three cases, the mutation was found on the β1 sheet of the first kinase domain, corresponding to amino acids 610–615. In one patient (M191, p.Leu601_Glu611dup), the duplication alters both the JM domain and the first residues of the β1 sheet in TKD. In the other two patients (M126: p.Leu610_Glu611ins20 and M169: p.Phe612_Gly613ins37), the integration occurred exclusively in the region of the β1 sheet of the first kinase domain. ITDs affecting the β1 sheet represent 22.2% of the ITD patients in this study, very similar to the frequency found in other series. No ITDs affecting different regions of the TKD were observed, although they have been described in a proportion of ITD patients (4).

In one patient, the in-frame loss of three nucleotides, producing Asp600 deletion in the JM domain, was observed. That mutation had a low VF. It does not correspond to the classic insertion/duplication occurring in the JM domain and has not been previously reported. It has been suggested that even a minimal mutation would be able to disrupt the intrinsic negative regulatory effects of the JM domain in preventing dimerization without ligand stimulation and would be sufficient to lead to auto-phosphorylation in *cis* (32). It could be hypothesized that this small deletion originates gain of function of the *FLT3* receptor, similar to ITD involving a more significant number of amino acid residues.

Revealing the heterogeneity of ITD mutations seems to be important in the clinical setting since it could be one of the factors leading to variability in the treatment response of the *FLT3*–ITD AML patients. Location of the ITD in *FLT3* influences the sensitivity to tyrosine kinase inhibitors as well as disease progression in mice (33). TKD-ITDs have been associated with a worse survival prognosis and to chemotherapy resistance in comparison with those with JM-ITD (30). In this study, only two patients with ITD-TKD were found; both had a high mutational burden. One of them had the highest mutational burden observed in our study (1.85 in patients M169). Marhäll et al. (34) showed that TKD-ITD and JM-ITD mutations display a similar oncogenic potential and that it is higher than the one from the D835Y point mutation located in the activation loop of the TKD.

*FLT3*-TKD mutations represent 47.4% (9/19) of the *FLT3*^POS^ cases, and 11.25% (9/80) of the total of patients, a higher prevalence than the 7.7% reported for AML adult patients (35). Six different mutations were observed; two of them were not listed in the COSMIC database (as of June 2020): p.Tyr842Ser, and, p.Met837del. Mutations within the activation loop of the second TKD were the most frequent (five of nine TKD cases). Asp835Tyr, considered the most common substitution in position 835, was found in only three patients, while Asp835His was identified in the other two cases.

*FLT3*-Asp835 mutations lead to constitutive activation of *FLT3* (36) and have been reported in 7–14% of AML (3), 3% of myelodysplastic syndromes, and 3% of acute lymphoblastic leukemia cases (37). Other reported substitutions, including Asp835Val, Asp835Glu, and Asp835Asn, were not found in our patients. The activating mutation p.Ile836del, identified in one patient, was described for the first time by Thiede et al. (35) in 13/979 AML adult patients.

The mutation p.Tyr842Ser was identified in one patient with VF = 26%. p.Tyr842Cys affecting the same codon has been associated with resistance to midostaurin, sunitinib, sorafenib, lestaurtinib, KW2449, and AGS324 (38). The main challenge with *FLT3* inhibitors and other target therapies is to overcome primary and secondary resistance. The possibility that Tyr842Ser could be related to primary resistance should be considered in the clinical setting. Since none of our patients were treated with *FLT3* inhibitors, the possible role of this mutation in response cannot be evaluated.

In an extensive series of pediatric AML patients, Bolouri et al. (12) reported mutations not found in adults with AML. New *FLT3* mutations affecting residues 451, 444, 491, 676, 680, and 941 were described, some of them with functional implications and influencing response to conventional therapy (20). If these mutations exist in our patients, they could not be detected due to target sequencing strategy limitations. Only mutations located between amino acids 534–647 and amino acids 807–847 could be detected. A full gene sequencing strategy is the best option to explore somatic mutations in *FLT3* and other genes too.

Two patients (22.2% of the *FLT3*^POS^ group) presented a second mutation at a lower VF, suggesting the presence of additional clones at diagnosis. A similar observation was reported by Kottaridis et al. (39) who found more than one mutation in 23% of *FLT3*–ITD AML patients, with up to five different *FLT3*–ITD clones of various sizes, insertion sites, and ARs identified. A worse OS has been observed in patients with more than one clone (39).

### Analysis of the Prognosis Impact of *FLT3* Mutation

OS was analyzed considering ITD and TKD mutations separated; both groups of patients showed lower survival than did the *FLT3*^NEG^ group (Figure 2). The influence of TKD or ITD on prognosis was very similar; no significant difference in OS between both groups of patients was observed.

The *FLT3*–ITD mutations have been previously related to a poor outcome in both adults (40, 41) and pediatric patients (42, 43). Wu et al. (44) performed a meta-analysis, including 1661 pediatric patients with AML. Patients with *FLT3*–ITD mutation had an inferior OS [hazard ratio (HR) = 2.19 (1.60–3.01), *p* < 0.001] in comparison with patients without *FLT3* mutation. The impact of TKD mutations was not analyzed in this paper. The author suggested that *FLT3*–ITDs produce a significantly negative prognostic effect in pediatric patients with AML.

There is no clear explanation of why *FLT3*–ITD could have such a negative impact. *FLT3*–ITD mutations contribute to increased production of reactive oxygen species (45), which leads to DNA oxidative damage, increases DNA double-strand breaks, and mistakes in the repair mechanisms starting a cycle of genomic instability. The existence of genomic instability leading to a cytogenetic evolution in AML is supported by the acquisition of new structural chromosomal abnormalities by leukemic cells between diagnosis and relapse. McCormick et al. (46) showed that cytogenetic evolution was more frequent among *FLT3*–ITD AML patients. Ten out of 14 *FLT3*–ITD AML patients acquired new cytogenetic and structural chromosomal abnormalities, compared with seven out of 21 *FLT3* wild-type AML patients. This observation has been confirmed by others analyzing *FLT3*–ITD AML patients at relapse (47). Other genomic features supporting genomic instability in *FLT3*–ITD patients have been shown, including microsatellite instability (48, 49), shorter telomeres (50), and high frequency of somatic mutations (51). We speculate that acquisition of additional poor-prognosis mutation is favored by the presence of genomic instability in *FLT3*–ITD patients and might be one of the factors contributing to the detrimental effect of *FLT3* mutations on survival (52).

Genomic instability is a hallmark of cancer, and high rates of genomic instability are present in other myeloid malignancies containing activated tyrosine kinase pathways, such as BCR/ABL in chronic myeloid leukemia, JAK2 in myeloproliferative neoplasias, and RAS mutations in myelodysplastic syndromes (45).

The effect of TKD mutations on prognosis has been mainly evaluated in adult patients and is considered controversial (3). Mead et al. (53) showed that patients with *FLT3*-TKD have a significantly more favorable prognosis than *FLT3*–ITD AML patients. In contrast, Whitman et al. (54) found that *FLT3* D835/I836 mutations were associated with poor disease-free survival and a distinct gene-expression signature among younger adults. Our results suggest that both *FLT3*-TKD and ITD mutations have a similar negative effect on OS in pediatric patients. Still, additional factors, like cytogenetic background, mutations in other genes, and treatment, may be contributing to the negative outcome in the TKD group. For example, the number of patients no-APL is significantly higher in TKD than in the ITD group (77.8% TKD vs. 30% ITD; *p*_Fisher_ = 0.046). Patients with other FAB subtypes had OS significantly lower than the APL group, treated with the PETHEMA-APL-05 (see Supplementary Figure 1B).

When the OS was considered, regardless of the subtype of *FLT3* mutation, patients with *FLT3* mutation had significantly lower OS than the *FLT3*^NEG^ patients, supporting that constitutive activation of the *FLT3* receptor, for any subtype of mutation, predisposes to high-risk disease. The negative effect of an *FLT3* mutation was additionally supported by the analysis in the APL group of patients. All APL patients were treated with the same protocol. However, APL-*FLT3*^POS^ patients had significantly worse OS that M3-*FLT3*^NEG^ patients. Other studies have shown an adverse effect of mutated *FLT3* in APL patients. Analyses of 205 adults and children found a significantly higher risk of death among APL patients with *FLT3*–ITD than in those without (HR = 11.74; 95% CI = 1.03–134.5), a reduced OS was also observed (15). Additionally, a higher relapse rate and a more reduced post-relapse survival have also been reported in the APL *FLT3*–ITD as compared with patients with APL *FLT3* wild-type (55, 56).

Both subtypes of *FLT3* mutation yield proteins that spontaneously dimerize, bypassing ligand-mediated activation. When they were transfected into murine cell lines, factor-independent growth originated (57). The analysis of 979 AML adult patients showed that both aberrations are also associated with similar clinical features: higher WBC and higher numbers of bone marrow blasts (35).

The impact of constitutive *FLT3* activation has been evaluated in several murine models. *FLT3*–ITD expression originated a myeloproliferative disorder having different phenotypes and severity depending on the murine model. In a bone marrow transplant model, a fatal myeloproliferative neoplasm occurred (57), while in a knock-in *FLT3*–ITD model, a less severe human chronic myelomonocytic leukemia like disease resulted. *FLT3* constitutive activation alone does not produce a full AML genotype; additional cooperating genetic alterations are needed in human and mouse for AML development (58).

*FLT3*–ITD is not expressed to the same level in all patients; differences in expression measured using the *FLT3*–ITD to wild-type AR also impact prognosis (3). It has been retrospectively shown that patients with a higher *FLT3*–ITD mutant-to-wild-type ratio have significantly worse outcomes than patients with a lower ratio, being 0.4 the cut-point in children and 0.5 in adults (59). In our ITD patients, the allelic burden ranged from 0.02 to 1.85; it was higher than 0.4 in 66.7% (6/9) of them. This study did not find that the mutational burden influences OS in the *FLT3*–ITD group or the whole *FLT3*^POS^ group. A significant limitation is the small sample size impairing the statistical power of the analysis. The role of the *FLT3* AR on the patient's outcome needs further clarification in prospective analysis with larger number of patients.

It has been suggested that when the *FLT3*–ITD AR is low, the presence of NPM1 mutation may mitigate the adverse prognostic effects of the *FLT3*–ITD mutation (60). However, all *FLT3*^POS^ patients had wild-type *NPM1*. Mutation in this gene was identified only in one of the cases (unpublished data), supporting the observation that this alteration is more common in adults than in pediatric AML patients (8).

Several limitations for this study must be considered: the results were not based on a randomized controlled trial but a retrospective analysis of a heterogeneously treated group of patients coming from eight different institutions. Patients were treated according to four different protocols that had different effects on the outcome. Besides, cytogenetic/fluorescence *in situ* hybridization (FISH) characterization, considered an independent prognostic factor in AML, was not performed in these patients. This study was not able to assess the potential effects of chromosomal aberration and mutations in other genes and its possible interaction with *FLT3* alterations. Since a full *FLT3* gene sequencing was not performed, mutation occurring outside exons 13–15 and exon 20 were not detected. Therefore, the frequency of *FLT3* mutation in Mexican pediatric AML patients may be even higher than the one obtained in the present work.

This is the first study evaluating the *FLT3* mutational profile in pediatric *de novo* AML Mexican patients and its impact on the prognostic. The results suggest that *FLT3* mutation could be considered as an independent biomarker for high-risk disease in pediatric AML patients and highlights the need for testing *FLT3* routinely in the clinical setting. A risk-adapted management decision may help to improve the clinical outcome in AML patients, similar to what has been achieved in adult AML patients.

The trend toward better response rates has seen in patients who received chemotherapy as first-line therapy in combination with *FLT3* inhibitors compared with those who did not receive it (3). Stone et al. (61) reported the results of an international randomized phase III study, including adult AML patients. Induction therapy (daunorubicin and cytarabine) and consolidation therapy (high-dose cytarabine), plus either midostaurin or placebo, revealed improved 4-year survival in the midostaurin arm compared with the placebo. These results were independent of allelic burden (high vs. low) or the type of *FLT3* mutation present (ITD or TKD). The benefit of midostaurin was observed among patients who underwent transplantation during the first remission but not among those who underwent transplantation at a later time (61). Even after intensive chemotherapy and stem cell transplant, *FLT3*–ITD is an adverse prognostic factor that remains prognostically relevant (3).

## Conclusions

A mutant *FLT3* is present in approximately one out of four pediatric AML patients in Mexico, and it is related to adverse outcomes. OS was impaired even in the APL subgroup that nowadays has higher complete remission and cure rates than other FAB subgroups due to more efficient therapeutic combinations.

All patients with newly diagnosed AML should have the *FLT3* tested using whole-genome sequencing approach better than a specific exon-target enrichment method to be able to detect some clinically relevant children with particular mutations. Earlier identification of *FLT3* mutational status will allow identification of patients with a more aggressive disease that could benefit from modification in the treatment strategy, including entering clinical trials, receiving focus therapy, or be considered for bone marrow transplant.

Increasing awareness about the importance and clinical utility of *FLT3* testing in AML is the first step to make *FLT3* testing part of routine diagnostic workups for these patients in Mexico.

## Data Availability Statement

The raw data supporting the conclusions of this article will be made available by the authors, without undue reservation.

## Ethics Statement

The studies involving human participants were reviewed and approved by National Institute of Genomic Medicine, Mexico City, Mexico (number of approval 28-2015-1). Written informed consent to participate in this study was provided by the participants' legal guardian/next of kin.

## Author Contributions

CM obtained the results, analyzed data, and drafted and approved the manuscript. KC, LLF, MJ, AMR, and BV obtained and validated the results and approved the manuscript. HF validated the results, analyzed statistical data, and approved the manuscript. JN, EJ, VB, JT, JF, JAM, MM, AMS, LE, JP, RE, LVF, RA, MP, OS, HR, AR, MD, EG, PG, and JMM collected clinical data, cared for patients, and approved the manuscript. MD, EG, and ER, validated the results and approved the manuscript. CA carried out the general oversight and the acquisition of funds, designed the study, obtained and validated the results, analyzed the data, and drafted and approved the manuscript. All authors contributed to the article and approved the submitted version.

## Conflict of Interest

The authors declare that the research was conducted in the absence of any commercial or financial relationships that could be construed as a potential conflict of interest.
