# Supplementary material for: Profiling FLT3 Mutations in Mexican Acute Myeloid Leukemia Pediatric Patients: Impact on Overall Survival
Source: Front Pediatr. 2020 Sep 16;8:586. doi: 10.3389/fped.2020.00586 (PMC7525023; doi:10.3389/fped.2020.00586)
Supplement: Supplementary file 1 [file Data_Sheet_1.docx]

***Supplementary material***

1. **Supplementary Data**

The genomic data underlying of this study can be found in the COSMIC site, the Catalog of Somatic Mutations in Cancer (<https://cancer.sanger.ac.uk/cosmic>).

The temporary accession number is: Cosmic identifier COSP47990

1. **Supplementary figures and tables**
   1. **Supplementary Table**

Supplementary Table 1. Treatment protocols assigned to AML pediatric patients, according to FAB subtypes.

| \| ^a^FAB classification \| Treatment protocols \| \| \| \| \| \| --- \| --- \| --- \| --- \| --- \| --- \| \| ^b^BFM-1998 \| ^c^NOPHO-AML93 \| BFM-2001 \| ^d^PETHEMA-APL05 \| *Total* \| \|  \| *N=25* \| *N=20* \| *N=6* \| *N=29* \| *80* \| \| M0 \|  \| 1 \|  \|  \| *1* \| \| M1 \| 4 \| 5 \|  \|  \| *9* \| \| M2 \| 10 \| 11 \| 6 \|  \| *27* \| \| M3 \|  \|  \|  \| 29 \| *29* \| \| M4 \| 9 \| 3 \|  \|  \| *12* \| \| M5 \| 1 \|  \|  \|  \| *1* \| \| M6 \| 1 \|  \|  \|  \| *1* \| |
| --- | --- | --- | --- | --- | --- | --- | --- | --- | --- | --- | --- | --- | --- | --- | --- | --- | --- | --- | --- | --- | --- | --- | --- | --- | --- | --- | --- | --- | --- | --- | --- | --- | --- | --- | --- | --- | --- | --- | --- | --- | --- | --- | --- | --- | --- | --- | --- | --- | --- | --- | --- | --- | --- | --- | --- | --- | --- | --- | --- |

Abbreviations: ^a^*FAB: French-American-British;* ^b^BFM: Berlin-Frankfurt-Münster, ^c^NOPHO: Nordic Society of Pediatric Haematology and Oncology, ^d^PETHEMA: Spanish Program of Treatments in Hematology, N: number of patients

- 1. **Supplementary Figures**


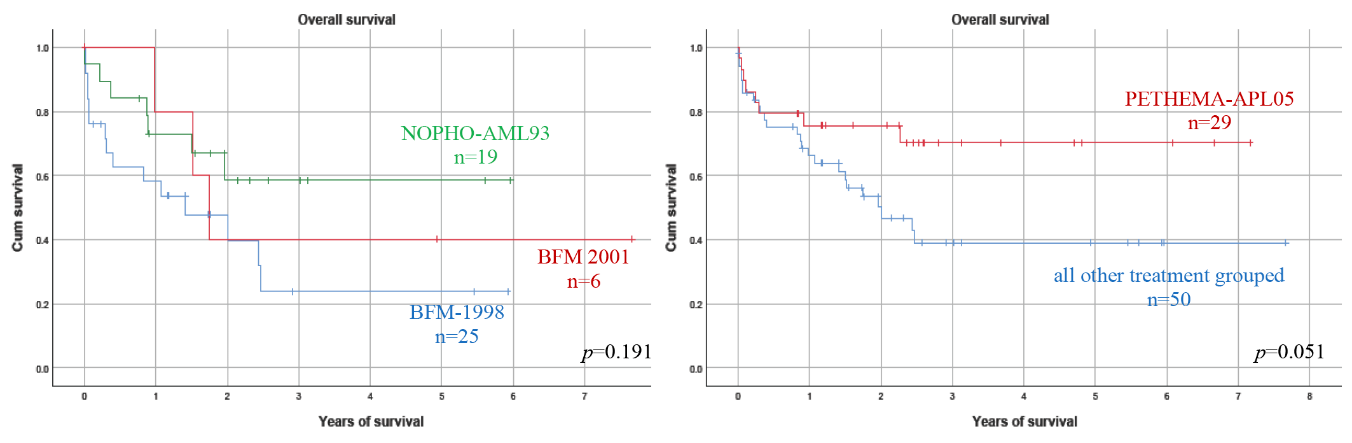


Supplementary Figure 1. Kaplan-Meier curves for overall survival according to treatment protocol **(A).** Overall survival for patients treated with BFM-1998, BFM-2001 (Berlin-Frankfurt-Münster), or NOPHO-AML93 (Nordic Society of Pediatric Haematology and Oncology). BFM-1998 treated patients had the worst OS and NOPHO-AML-93 the best, but differences were not statistically significant. **(B).** Overall survival in the PETHEMA-APL05 treated group, corresponding to APL (M3), is significantly higher than the average OS in the group of patients receiving any other treatment (long-rank test p=0.05)


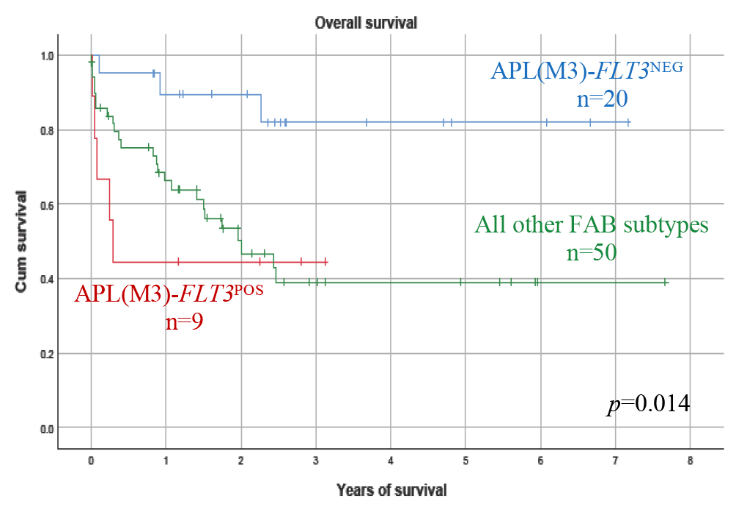


Supplementary Figure 2. Kaplan-Meier curves for overall survival in APL(M3) patients by *FLT3* status. All no-M3 (“all other FAB subtypes”) patients were grouped and included for comparison. Significant differences exist in OS; the APL-*FLT3*^NEG^ patients had the best OS as compared with APL-*FLT3*^POS^ patients or “all other FAB subtypes.” These last two groups have a very similar OS.
